# Thermal spiral inductor using 3D printed shape memory kirigami

**DOI:** 10.1038/s41598-022-26923-3

**Published:** 2022-12-23

**Authors:** Yelim Kim, Ratanak Phon, Heijun Jeong, Yeonju Kim, Sungjoon Lim

**Affiliations:** School of Electrical and Electronics Engineering, Chung-Ang University, Seoul, 06974 Republic of Korea

**Keywords:** Electrical and electronic engineering, Structural materials

## Abstract

Spiral inductors are required to realise high inductance in radio frequency (RF) circuits. Although their fabrication by using micro-electrical–mechanical systems, thin films, actuators, etc., has received considerable research attention, current approaches are both complex and expensive. In this study, we designed and fabricated a thermal spiral inductor by using a three-dimensional (3D) printed shape-memory polymer (SMP). The proposed inductor was inspired by kirigami geometry whereby a two-dimensional (2D) planar geometric shape could be transformed into a 3D spiral one to change the inductance by heating and manually transform. Mechanical and electromagnetic analyses of the spiral inductor design was conducted. Hence, in contrast with the current processes used to manufacture spiral inductors, ours can be realised via a single facile fabrication step.

## Introduction

Shape-memory polymers (SMPs) have interesting features such as shape programming and self-recovery in response to external stimuli such as light, moisture, pH changes and heat^1,2^. With advances in materials technology, SMPs have shown promising applicability in both science and engineering. For instance, they have been utilised in the fields of sensors^3^, biomedicines^4^, actuators^5^ and self-healing^6^. Recently, SMPs have caught the interest of many researchers and have been used in various electromagnetic devices, such as frequency memorizing microstrip monopole antennas that can switch between two different operating frequency modes^7^. Since the shape of SMPs can be reversibly altered via mechanical transformation, they have been used in many reconfigurable multifunctional electromagnetic and electronic devices^8–10^. However, SMPs have a drawback in that the memory function is only singular. Specifically, they can form a predetermined shape in response to heating and/or mechanical stress and then automatically recover to their original shape by just heating. This is a major problem that requires further research to improve the performance and functionality of SMPs.

Three-dimensional (3D) printing is an additive-manufacturing technique that is used by many scientists and engineers to rapidly produce low-cost prototypes^11,12^. Fused deposition modelling (FDM) is the most widely used 3D printing method that creates objects by melting a plastic or dielectric filament and then depositing it in layers. It has been used in various scientific and industrial processes to construct many items with complex shapes. In addition, it can be used to easily create functional and/or shape-programmable structures by integrating various functional materials using 3D multi-material techniques. Although the SMPs have been applied in several electronic, electromagnetic and other devices^3,7–10,13,14^, a 3D-printing based thermal spiral inductor using SMP has not previously been reported.

Herein, we propose an SMP-based 3D-printed spiral inductor that is shape-programmable via external heating. Conventionally, a reconfigurable spiral inductor can be achieved by using microelectromechanical systems technology^15,16^ or printed circuit board processing where the spiral shape is obtained by connecting multilayers with vias. In addition, reconfigurability is achieved by selecting the number of turns^17,18^. Nevertheless, these conventional techniques are complex and costly. In the present study, the proposed spiral inductor was fabricated by using a 3D-printing-based SMP that provides the feasibility to program any predetermined shape and return to the original shape via mechanical transformation by heating and then cooling to room temperature, respectively. The original 3D-printed shape can be recovered by heating and then cooling to automatically return it to its original shape. We numerically and experimentally examined the properties of the programmed and restored shapes, including their inductance, Q-factor values and resonant frequencies. Since the cost and time required to fabricate the proposed spiral inductor are low and its functionality is dependent on the temperature, we believe that it could be used in sensors and rapidly fabricated low-cost prototypes.

## Results and discussion

### Characteristics of the SMP

**Figure 1:**
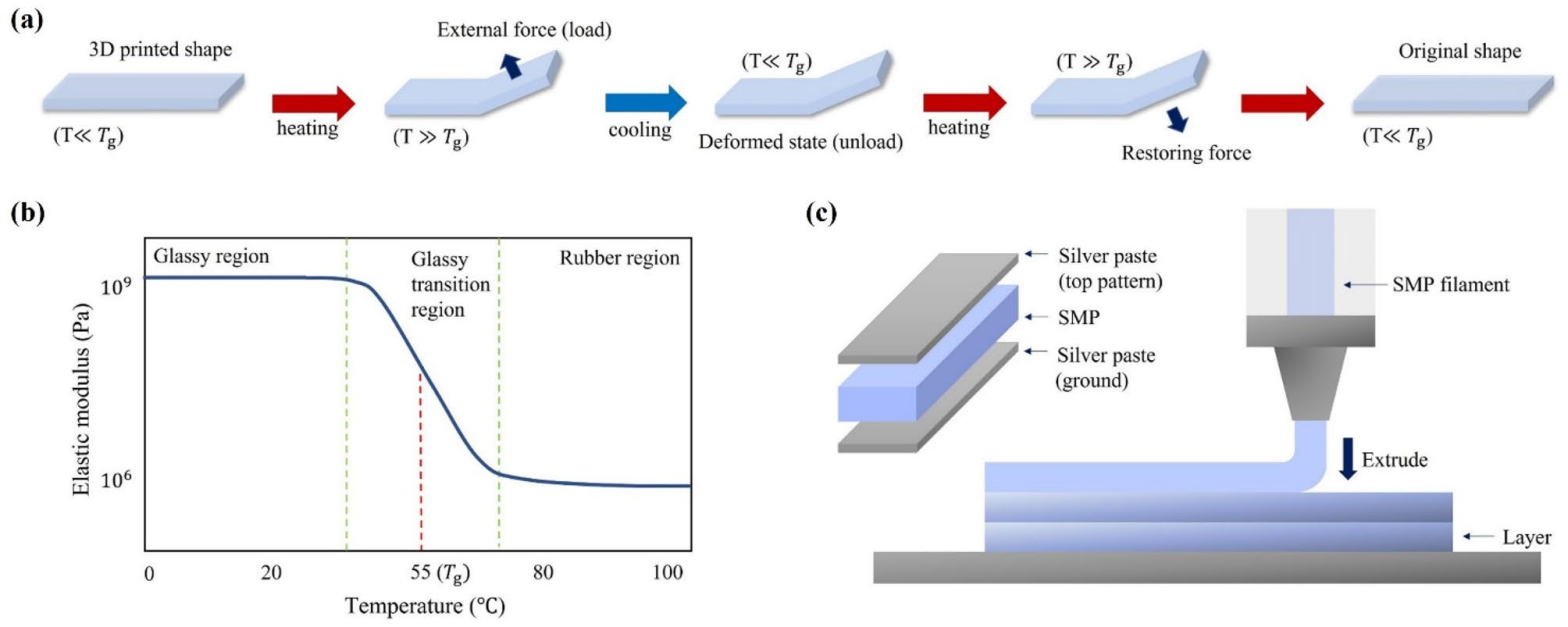
The mode of operation and production of an SMP structure. (**a**) A schematic of the transformation and recovery steps of an SMP structure. (**b**) Elastic modulus vs. temperature of the SMP filaments showing the glass-transition temperature (*T*_*g*_). (**c**) FDM 3D printing of the designed SMP-based spiral inductor.

SMP filaments from SMP Technologies Inc. with a dielectric constant of 3 and loss tangent *δ* of 0.03 were used to fabricate the thermal spiral inductor. Other properties of the SMP material are provided in the following section. ELCOAT silver resin paste with a conductivity of approximately 10^6^ S/m was used for the conductive layer in the spiral inductor^19^. In general, the shape of an item fabricated with SMP formed by applying external heat and using external force will reacquire its shape after cooling. The transforming and restoring temperatures are determined by using the glass-transition temperature (${T}_{\mathrm{g}}$), which is the temperature transition point between the rigid glassy and soft rubbery states. Figure 1a shows the four steps comprising the thermally transformed SMP filament shape recovery cycle.

1. The original 3D-printed structure is transformed when $T>{T}_{\mathrm{g}}$.
2. An external force is then applied to transform the structure while heating.
3. The external force is continuously applied to retain the new shape as the heat is reduced to room temperature and is then removed while the new shape remains in place.
4. Reapplying external heat ($T>{T}_{\mathrm{g}}$) restores the new shape to the original shape.

Figure 1b shows elastic modulus changes depending on the applied temperature of the SMP material; it can be seen that the elastic modulus value decreases as the temperature was increased from approximately 35 to 75 °C. In this work, we used SMP filaments with a *T*_g_ of 55 °C and a measured density of 1073.596 kg/m^3^. The SMP is in the glassy state when the external temperature is lower than the room temperature and in the rubbery state when the external temperature is higher than 80 °C. The SMP has an elastic modulus value of 10^9^ Pa in the glassy state (i.e., it is still a hard solid) and an elastic modulus of 10^6^ Pa in the rubbery state (i.e., it is flexibly transformable)^20,21^.

We employed the additive-manufacturing method FDM 3D-printing to build the melting filament layer by layer in the desired geometry. Figure 1c shows the manufacturing process to fabricate the proposed SMP-based spiral inductor. The heated nozzle melted the SMP filament and extruded it layer by layer. In addition, the attached filament layer was cooled by using a fan and became hard. Silver paste was applied to the top and bottom surfaces using a brush to provide its electrical characteristics.

**Figure 2:**
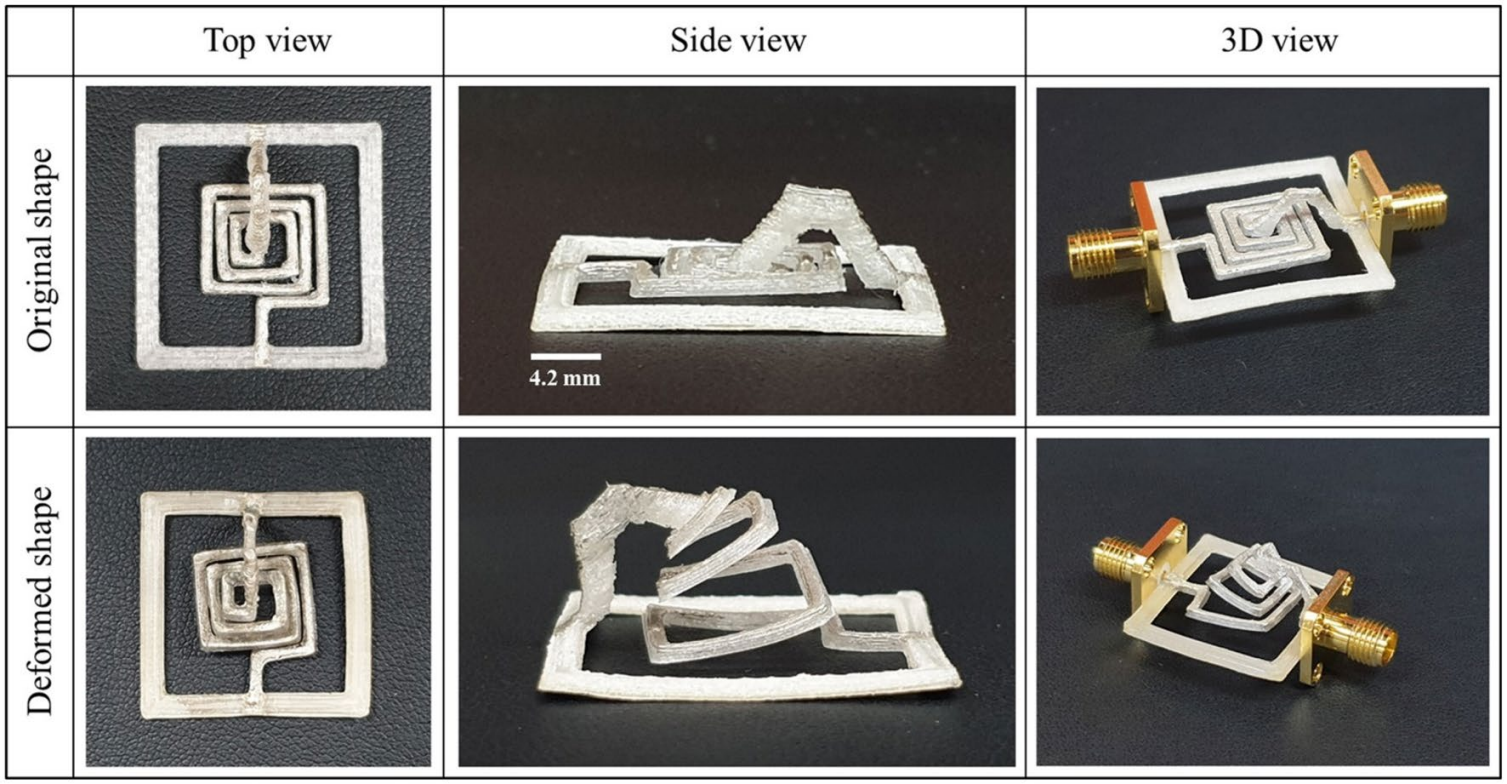
Images of the fabricated inductor from various viewpoints.

Figure 2 shows images of the fabricated sample in the original and transformed shapes from various viewpoints. The planar spiral line areas were transformed into a conical shape. The fabricated sample was transformed by hand and heat was applied using a hot plate. An SMA connector was used to measure the inductance of the device.

### Investigation of the 3D-printed thermal spiral inductor

In general, a spiral inductor is fabricated with a via or vertical shape air bridge. However, the FDM method has a manufacturing limitation when fabricating a horizontal structure in air as the structure needs to be supported. Thus, we designed an inclined air bridge, the angle of which was determined via fabrication experiments.

**Figure 3:**
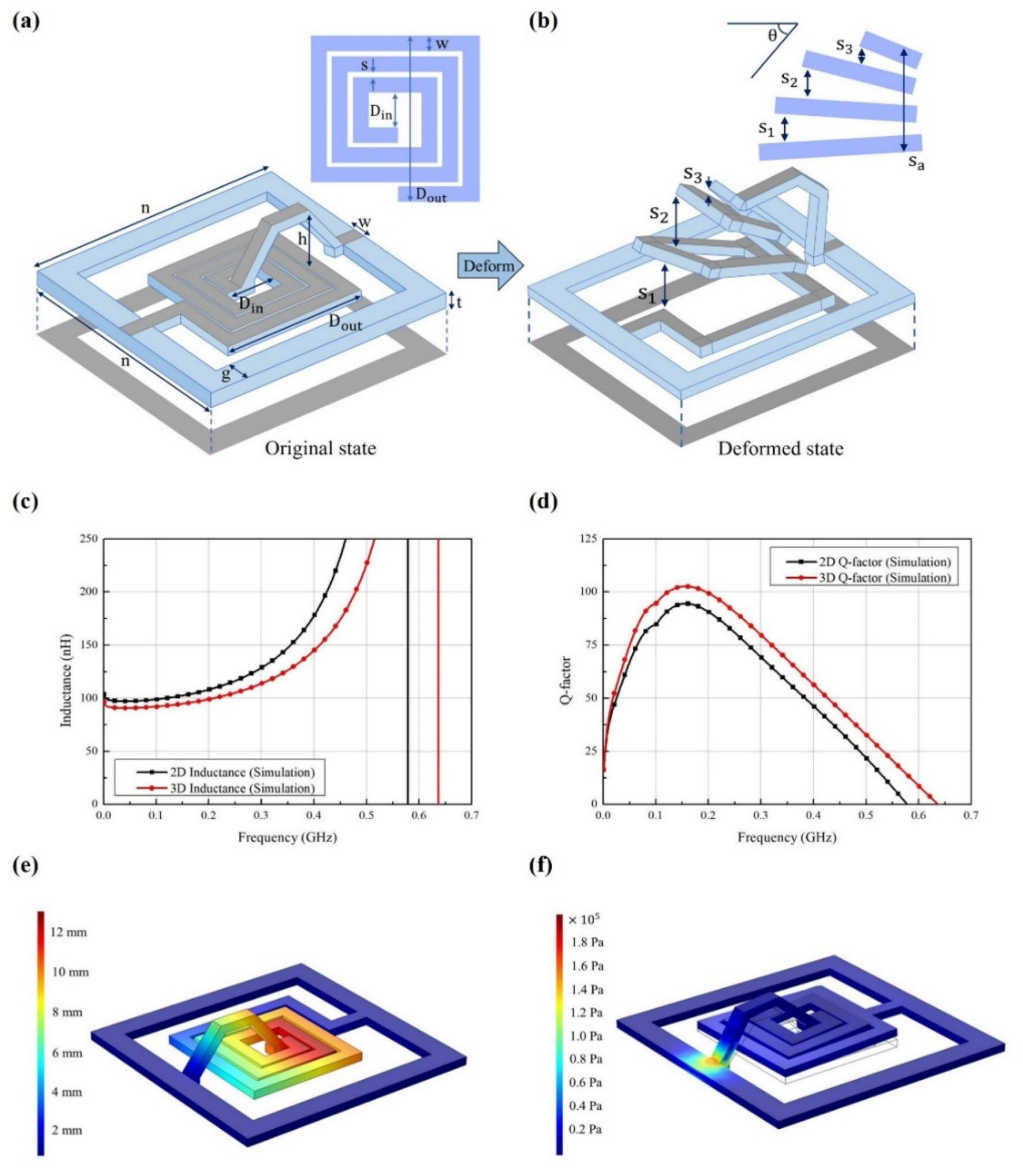
Schematics of the proposed thermal spiral planar inductor in its (**a**) original 2D planar rectangular shape and (**b**) 3D transformed spiral shape. The simulated (**c**) inductance and (**d**) Q-factor of each shape. The simulated (**e**) total displacement and (**f**) stress in the original shape. *D*_*in*_, the internal diameter of the inductor (2.9 mm); *D*_*out*_, the external diameter of the inductor (14.2 mm); *n*, the outer length and width of the inductor (25.2 mm); *h*, the height of the air bridge (6 mm); *w*, the width of the inductor spiral (1.4 mm); *g*, the width of the surrounding square grid (2.3 mm); *S*_*a*_, the height of the spiral (12.5 mm); *S*_1_–*S*_3_, the distances between the inductor lines in the spiral (3.5, 4.5 and 0.5 mm, respectively); *a*, the angle of transformation (30°).

Figure 3 shows details of the design parameters of the fabricated sample in the original and transformed shapes. Figure 3a shows the design parameters for the original 2D planar rectangular shape of the proposed inductor. The performance of a spiral inductor is determined by the number of turns in, as well as the inner and outer diameter of the spiral range. The designed inductor is surrounded by a square grid that has two functions: as the ground and as a fixed frame. The inductor is surrounded by a microstrip line with an input impedance of around 70 Ω. The inclined air bridge fabrication also affects the inductor width. The air bridge design parameters were determined for a single-step fabrication process without any post-processing. The air bridge has height *h* = 6 mm, an angle from the horizontal of 55$^\circ$ and a total length of 16.1 mm. In addition, the distance between the lines is 0.3 mm and the internal diameter (${D}_{\mathrm{in}}$) and external diameter (${D}_{\mathrm{out}}$) of the inductor are 2.9 and 14.2 mm, respectively. The inductance of the original-shaped inductor can be calculated by using a general spiral planar inductor inductance equation as follows:

1 $$ L_{{{\text{pl}}}} = \mu N^{2} r_{{{\text{avg}}}} \left[ {\ln \left( {\frac{2.46}{{R_{{\text{r}}} }}} \right) + 0.2R_{{\text{r}}} } \right], $$

where $N$ is the number of turns in the spiral inductor, ${r}_{\mathrm{avg}}=\left({D}_{\mathrm{in}}+{D}_{\mathrm{out}}\right)/2$ and ${R}_{\mathrm{r}}=\left({D}_{\mathrm{out}}-{D}_{\mathrm{in}}\right)/\left({D}_{\mathrm{out}}+{D}_{\mathrm{in}}\right).$

When heat is applied to the spiral planar inductor and force is applied perpendicular to the air bridge plane, then the parameters change, as shown in Fig. 3b. The transformed shape is similar to a quadrangular pyramid. The total height ${s}_{\mathrm{a}}$ = 12.5 mm; the distances between the inductor lines ${s}_{1}$, ${s}_{2}$ and ${s}_{3}$ = 3.5, 4.5 and 0.5 mm, respectively; and angle of transformation $\alpha$ = 30°. The inductance of the transformed inductor can be approximated to that of a conical inductor as follows:

2 $$ L_{{{\text{co}}}} = \sqrt {L_{{{\text{pl}}}}^{2} \cos^{2} (\alpha ) + L_{{{\text{cy}}}}^{2} \sin^{2} (\alpha )} , $$

where $\alpha$ is the angle of a conical inductor and ${L}_{\mathrm{cy}}$ is the inductance of a cylindrical inductor with has same radius size determined as

3 $$ L_{{{\text{cy}}}} = \frac{{10\pi N^{2} r_{{{\text{avg}}}}^{2} }}{{9D_{{{\text{in}}}} + 10s_{a} }}. $$

In addition, the Q-factor of the inductor can be expressed as

4 $$ Q = \frac{2\pi fL}{R}, $$

where $f$ is the frequency, $L$ is the inductance and $R$ is the resistance^22,23^.

We used Ansys high-frequency structure simulator (HFSS) to investigate the fabricated kirigami-inspired structure operating as a spiral inductor with silver paste conductivity. Figure 3c shows that the simulated inductance by the transformed and restored shapes in the 250 MHz range increased from 95 to 102.7 nH, respectively, and that the self-resonant frequency decreased from 637 to 578 MHz, respectively. Figure 3d shows that the maximum Q-factor decreased from 102.6 to 94.5, respectively. Deforming the structure caused the mutual inductance and capacitance between the inductor lines to decrease, which affected the resonant frequency and Q-factor^24–26^.

We also simulated the transformed shape to investigate its physical characteristics by using a general constitutive SMP based on SMP materials used in various constitutive models and mechanical applications such as actuators. We used COMSOL Multiphysics software to simulate the mechanical transformation properties for the proposed 3D-printed spiral inductor in which the partial differential equations are used to simulate the physical phenomena. The spiral inductor structure in HFSS was extracted and imported to COMSOL Multiphysics and subsequently analysed. We used Young’s modulus, which depends on three distinct states based on the temperature: glassy (*T* < *T*_g_), glass-transition (*T*_g_) and rubbery (*T* > *T*_g_). The SMP used in this work had a Young’s modulus value of 1 MPa, a Poisson’s ratio of 0.4 and a density of 1073.596 kg/m^3^. The prepared SMP filament had an approximately linearly decreasing elastic modulus with increasing temperature in the glass-transition region. The fabricated structure transformed at 75 °C under a force applied by hand in the perpendicular direction to the air bridge, which closed the surrounding line plane. The COMSOL Multiphysics program was used to simulate total displacement under the applied stress at 75 °C and SMP elastic modulus *E* = 10^6^.

Figure 3e,f show the simulation results for the inductor in the transformed shape: a total displacement of 12.5 mm occurred under a load of 0.03 N perpendicular to the air bridge. In addition, the maximum applied stress of $1.6\times {10}^{5}$ Pa was concentrated at the base of the air bridge.

**Figure 4:**
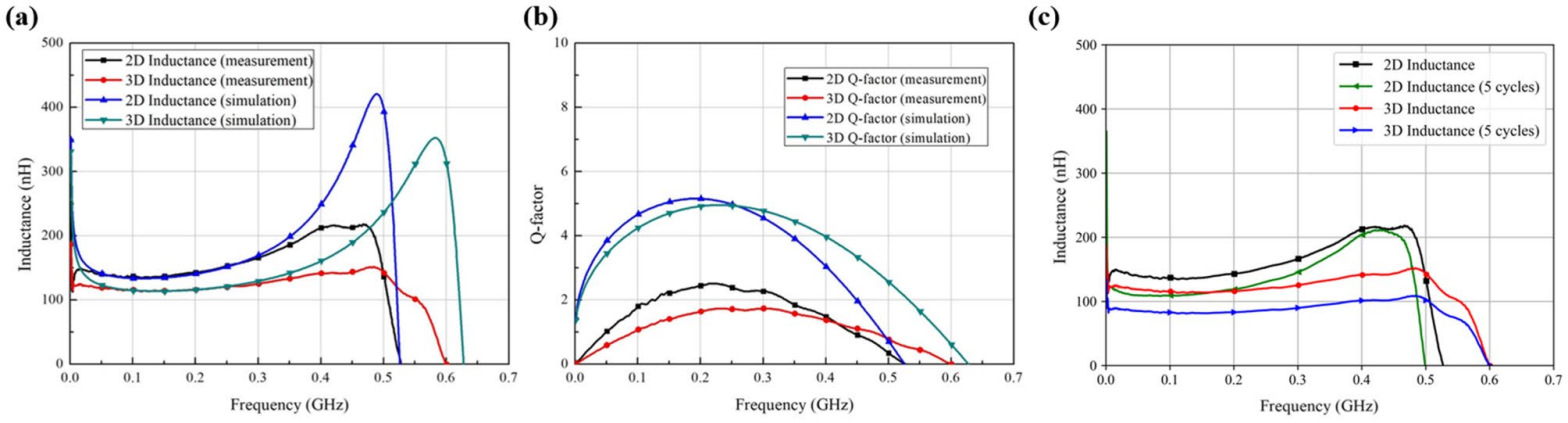
Measurements of the characteristics of the fabricated inductor: (**a**) inductance, (**b**) Q-factor, (**c**) after 5 times repeating cycles.

Figure 4 shows the measurement results of the characteristics of the fabricated thermal inductor using a vector network analyser (VNA). The results in Fig. 4a show that when considering the surface resistance, the measured inductance results were the same as the simulation results. As the transformed inductor was restored to the original 2D planar spiral inductor shape in the 250 MHz range, the average inductance changed from 117.3 to 140.9 nH (20.12%), respectively. Moreover, the self-resonant frequency decreased from 600 to 528 MHz, respectively. Figure 4b shows the measured maximum Q-factor increased from 1.73 to 2.52, respectively. In the measurement in Fig. 4b, we notice that the Q-factor is lower compared to the simulated result in Fig. 3d. This is due to the low conductivity of silver ink used in the fabrication. We found that the conductivity of this silver ink is approximately 15,000 S/m as compared between simulation and measurement in Fig. 4b. Figure 4c shows measured results of the thermal spiral inductor after 5 times repeating cycles. We notice that the inductance values of the sample is almost stable with a minor deviation. Although the simulated inductor is the same size as the fabricated sample, applying the silver past to the rough surface caused by using the FDM method reduced the surface conductivity. The angle of the transformed shape affected the Q-factor and parameters ${s}_{1}$, ${s}_{2}$ and ${s}_{3}$ (1.5, 5 and 2 mm, respectively). The experimental results indicate that the SMP material used in the 3D-printed structure can be applied to electromagnetic structures and sensor devices.

## Conclusions

We fabricated a novel SMP-based thermal spiral inductor fabricated by using FDM 3D-printing technology. Silver paste was applied to the top and bottom surfaces to provide the electrical characteristics of the proposed spiral inductor. The SMP-based thermal spiral inductor could be transformed by applying a force at 75 °C and returned to its original shape after cooling to room temperature. The fabricated inductor in both the original and transformed shapes had different inductances, Q-factor values and self-resonant frequencies. Restoring the original shape from the transformed shape increased the Q-factor and reduced the self-resonant frequency and inductance (the latter by 20.12%).

## Methods

### Fabrication and measurement of the 3D-printed inductor

The designed kirigami-inspired 3D structure was printed by using a 3DWOX 7X 3D printer. The SMP filament was purchased from SMP Technologies Inc. The nozzle and bed temperatures to achieve a clear sample were 210 and 100 °C, respectively, and the infill affecting the density was 20%. We coated ELCOAT P-100 silver paste with a bulk conductivity of 10^6^ S/m used as conductivity ink on the top and bottom planes by using a brush, which was allowed to dry to room temperature (25 °C) for 12 h. The resistance measured by using a multimeter was 1 Ω. In the next step, conductive epoxy CW 2400 was used to connect the fabricated inductor with the SMA connector because the SMP material distorts and melts at high temperatures. The 3D-printer had manufacturing errors in the *x-*, *y-* and *z*-directions, with average differences (fabricated sample size vs. designed sample size) of + 0.2 mm, + 0.1 mm and − 0.15 mm, respectively, which were compensated for in the fabrication.

To determine the value of inductance (*L*) and quality factor (*Q*) in the measurement, we measure the scattering parameter (S-matrix) of the proposed spiral inductor using VNA and then convert to admittance matrix (Y-matrix). The values of *L* and *Q* are determined from the following equation^27^:

5 $$ L = \frac{{{\text{Im}} \left( {\frac{4}{{Y_{11} + Y_{22} - Y_{12} - Y_{21} }}} \right)}}{2\pi f}, $$

6 $$ Q = - \frac{{{\text{Im}} (Y_{11} + Y_{22} - Y_{12} - Y_{21} )}}{{{\text{Re}} \left( {Y_{11} + Y_{22} - Y_{12} - Y_{21} } \right)}}. $$

## Data Availability

The datasets used and/or analysed during the current study available from the corresponding author on reasonable request.
